# Branched-chain amino acids and the risks of dementia, Alzheimer’s disease, and Parkinson’s disease

**DOI:** 10.3389/fnagi.2024.1369493

**Published:** 2024-04-10

**Authors:** Yidong Fu, Yue Wang, Huiming Ren, Xu Guo, Liyuan Han

**Affiliations:** ^1^Department of Rehabilitation Medicine, Ningbo No. 2 Hospital, Ningbo, China; ^2^School of Public Health, Medical College of Soochow University, Suzhou, China; ^3^Department of Clinical Epidemiology, Ningbo No. 2 Hospital, Ningbo, China; ^4^Center for Cardiovascular and Cerebrovascular Epidemiology and Translational Medicine, Ningbo Institute of Life and Health Industry, University of Chinese Academy of Sciences, Ningbo, China

**Keywords:** branched-chain amino acids, dementia, Alzheimer’s disease, Parkinson’s disease, correlation analysis

## Abstract

**Background:**

We aimed to examine the association between blood levels of Branched-chain amino acids (BCAAs) - specifically isoleucine, leucine, and valine - and the susceptibility to three neurodegenerative disorders: dementia, Alzheimer’s disease (AD), and Parkinson’s disease (PD).

**Methods:**

Based on data from the UK Biobank, a Cox proportional hazard regression model and a dose–response relationship were used to analyze the association between BCAAs and the risks of dementia, AD, and PD. We also generated a healthy lifestyle score and a polygenic risk score. Besides, we conducted a sensitivity analysis to ensure the robustness of our findings.

**Results:**

After adjusting for multiple covariates, blood concentrations of isoleucine, leucine, and valine were significantly associated with a reduced risk of dementia and AD. This association remained robust even in sensitivity analyses. Similarly, higher levels of isoleucine and leucine in the blood were found to be associated with an increased risk of PD, but this positive correlation could potentially be explained by the presence of covariates. Further analysis using a dose–response approach revealed that a blood leucine concentration of 2.14 mmol/L was associated with the lowest risk of dementia.

**Conclusion:**

BCAAs have the potential to serve as a biomarker for dementia and AD. However, the specific mechanism through which BCAAs are linked to the development of dementia, AD, and PD remains unclear and necessitates additional investigation.

## 1 Introduction

Neurodegenerative diseases, such as dementia, are a significant cause of disability and death worldwide (GBD 2015 Neurological Disorders Collaborator Group, 2017). Dementia is a persistent, acquired cognitive impairment caused by brain dysfunction and is classified into two types: dementia caused by non-neurodegenerative diseases, and dementia caused by neurodegenerative diseases, of which Alzheimer’s disease (AD) is the most common (James and Bennett, 2019). Parkinson’s disease (PD) is a progressive neurodegenerative disorder. A significant number of individuals with PD also develop dementia, with an incidence rate nearing 30% (Hanagasi et al., 2017).

Blood metabolites, which are small molecules that reflect the interplay of genetic and environmental factors and serve as the end-products of intricate cellular regulatory pathways, are considered reliable indicators of disease processes (Wang et al., 2019). One such group of metabolites is Branched-chain amino acids (BCAAs), including leucine, isoleucine, and valine, which are crucial for protein synthesis and require dietary intake. Studies have connected BCAA intake levels to numerous conditions, such as high blood pressure, atherosclerosis, coronary heart disease, heart failure, cancer, and insulin resistance (Grajeda-Iglesias and Aviram, 2018; Nie et al., 2018; Flores-Guerrero et al., 2019; Lim et al., 2020; Chevli et al., 2021). Interestingly, accumulating evidence demonstrating that BCAAs can trigger neurodegeneration and participate in the pathogenesis of neurodegenerative disorders (Yoo et al., 2022).

However, the existing literature on the association between BCAAs and dementia, AD, and PD mostly consists of conventional studies lacking sufficient population-based prospective studies. For example, a longitudinal study showed the concentrations of leucine and isoleucine progressively decreased with the progression of PD (Yan et al., 2022). Another study suggested that elevated plasma isoleucine levels were found to be associated with Alzheimer’s disease (Larsson and Markus, 2017). These aforementioned findings emphasize the necessity of further clarifying the association between BCAAs and dementia, AD, and PD.

Therefore, we used the UK Biobank data to investigate the associations between blood concentrations of leucine, isoleucine, and valine, and the risks of dementia, AD, and PD. We further examined whether a healthy lifestyle score based on four variables: smoking status, level of physical activity, diet, and alcohol consumption at baseline, and a polygenic risk score, could modify the association of blood concentrations of BCAAs with neurodegenerative disorders.

## 2 Methods

### 2.1 Sample

The UK Biobank is a prospective cohort study which aimed to collect data from a population-based sample of UK residents. Between March 13, 2006, and October 1, 2010, a total of 502,413 individuals were enrolled as participants and have been followed up since then. The complete study protocol for the UK Biobank is available to the public, and details regarding data collection have been previously described (Sudlow et al., 2015). To gather information, a touch-screen questionnaire survey was administered to participants at assessment centers located in urban areas across England, Wales, and Scotland. This survey included questions related to participants’ demographics, socio-economic status, lifestyle choices, and health. Furthermore, anthropometric measurements were taken during the assessment process.

Supplementary Figure S1 demonstrates the exclusion criteria applied in the present study. Participants were excluded if they had missing data on blood BCAA concentrations (*n* = 384,423), smoking status, drinking status, BMI, physical activity, income, and number of years of education (*n* = 49,628), or genetic data (*n* = 330). Ultimately, a total of 68,032 participants were included in the analysis. It is important to note that the UK Biobank obtained ethical approval from the Research Ethics Committee (Reference 11/NW/0382), and all participants provided electronic signed consent.

### 2.2 Endpoint

The endpoint of this study was to observe the occurrence of dementia, AD, or PD between the years 2019 and 2021. To determine the presence of these conditions, data from the UK Biobank were obtained, which included information from primary care records, hospital admissions, and death registries. The correlation of this data was necessary to establish the occurrence of dementia, AD, and PD. A diagnosis of any of these diseases was made based on the respective codes for neurodegenerative diseases provided in the 10th Revision of the International Statistical Classification of Diseases and Related Health Problems. Further details can be found in Supplementary Table S1.

### 2.3 Exposure

BCAA exposure, which is one of the metabolic biomarkers, was quantified using nuclear magnetic resonance spectroscopy by Nightingale Health. They measured these biomarkers in ethylene diamine tetra-acetic acid plasma samples collected from approximately 280,000 participants of the UK Biobank between 2019 and 2020. In this study, different concentration ranges were used to define low, medium, and high levels of isoleucine, leucine, and valine. Specifically, for isoleucine, concentrations less than 1.62 mmoL/L (Q1) were considered low, while concentrations ranging between 1.62 and 1.74 mmoL/L (Q2) were classified as medium, and concentrations greater than 1.74 mmoL/L (Q3) were classified as high. Similarly, for leucine, concentrations less than 1.95 mmoL/L (Q1) were considered low, concentrations ranging between 1.95 and 2.04 mmoL/L (Q2) were classified as medium, and concentrations greater than 2.04 mmoL/L (Q3) were classified as high. Finally, for valine, concentrations less than 2.26 mmoL/L (Q1) were classified as low, concentrations ranging between 2.26 and 2.34 mmoL/L (Q2) were classified as medium, and concentrations greater than 2.34 mmoL/L (Q3) were classified as high. Additional details regarding the BCAA exposure data can be found in Supplementary Table S2.

### 2.4 Covariates

We developed a comprehensive healthy lifestyle score (HLS) to assess the correlation between lifestyle choices and the risks of dementia, AD, or PD, utilizing previously identified risk factors for these conditions (Livingston et al., 2020; Zhao et al., 2020). The participants’ HLS was determined based on four key variables: smoking status, physical activity level, diet, and alcohol consumption. These variables were evaluated using a touch-screen questionnaire administered at baseline from 2006 to 2010. Smoking status was categorized by assigning 1 point to those who were either former smokers or had never smoked, and 0 points to current smokers. Physical activity level was determined by assigning 1 point to individuals engaging in a minimum of 150 min per week of moderate physical exercise or 75 min per week of vigorous physical exercise, and 0 points otherwise. Diet was assessed using three variables: fruit and vegetable intake, red meat intake, and processed meat intake. Participants received 1 point if their daily fruit and vegetable intake was equal to or greater than six portions, if their weekly red meat intake was less than seven portions, and if their weekly processed meat intake was less than four portions. Alcohol consumption was quantified in terms of the number of glasses consumed per week. This was calculated as the sum of red wine intake multiplied by 0.85, champagne and white wine intake multiplied by 0.85, beer and cider intake multiplied by 1.28, spirits intake multiplied by 0.75, and strong wine intake multiplied by 0.56. A score of 1 was allocated to individuals whose alcohol intake was less than 1 glass per week for women and fewer than 2 glasses per week for men. Otherwise, 0 points were assigned. The highest possible HLS was 4, and a score of 0–1 indicated an unhealthy lifestyle, a score of 2 reflected a moderately healthy lifestyle, and a score of 3–4 represented a very healthy lifestyle. For further details, please refer to Supplementary Table S3.

Polygenic risk scores (PRSs) were generated through a Bayesian meta-analysis of aggregated statistical data obtained from two sources: genome-wide relationship study (GWAS) data. These data were either solely derived from external GWAS data, resulting in what we refer to as standard PRS sets, or a combination of external and internal (i.e., UK Biobank) data, which we term enhanced PRS sets (Chouraki et al., 2016). The PRSs were categorized into low, medium, and high risk groups, representing the lowest, intermediate, and highest tertiles of genetic risk, respectively.

The age, sex, BMI, income, education level, race, smoking status, diabetes status, history of high blood pressure, stroke status, and lipid profile data of the participants were collected through touch-screen questionnaires and anthropometric measurements at baseline (2006–2010). BMI was calculated as the ratio of body weight (in kilograms) to height (in meters) squared. Considering the involvement of the Apolipoprotein E (APOE) gene ε4-allele gene as a genetic risk factor for late-onset AD, we determined the APOE genotype using the APOE single nucleotide variants (SNVs) rs429358 and rs7412. Participants with one or two copies of the ε4-allele gene were classified as APOE ε4 carriers, while those without any copies were classified as APOE ε4 non-carriers.

### 2.5 Data analysis

Continuous and categorical variables were presented as means ± standard deviations (SDs) and numerical values (percentages), respectively. Cox proportional hazard regression models were employed to estimate the associations between blood concentrations of BCAAs and dementia, and hazard ratios (HRs) and 95% confidence intervals (CIs) were calculated. Model 1 was adjusted for age, sex, ethnicity, income, work, education, alcohol consumption, smoking status, BMI, SBP, hypertension, and diabetes. Model 2 further adjusted for stroke, while model 3 additionally adjusted for HDL, LDL, TG, and TC. Restricted cubic splines were utilized to explore the dose–response relationships between blood concentrations of BCAAs and the risk of dementia. In addition, we conducted several sensitivity analyses to evaluate the robustness of the findings. Firstly, we excluded participants who developed dementia within the 2 years preceding the follow-up in order to minimize the impact of reverse causality. Secondly, we carried out the analysis with additional adjustment for blood concentrations of lipids (including high-density lipoprotein cholesterol, low-density lipoprotein cholesterol, total cholesterol, and triglycerides) and glucose in order to reduce potential residual confounding. To assess the relationship between blood concentrations of BCAAs and dementia in relation to PRS, HLS, age, BMI, education level, smoking and alcohol consumption status, and ApoE status, we conducted stratified analyses. All statistical analyses were performed using R software with a two-sided test, and a *p* value less than 0.05 was considered statistically significant.

## 3 Results

### 3.1 Baseline characteristics

The mean age of the participants in the study was 56.51 years (SD = 8.13 years). The majority of the participants were female (*n* = 34,197; 50.27%), Caucasian (*n* = 64,779; 95.22%), had received 15–20 years of schooling (*n* = 35,844; 52.69%), had a BMI of 25–30 (*n* = 29,402; 43.22%), had never smoked (*n* = 36,877; 54.21%), currently drank alcohol (*n* = 63,454; 93.27%), had a high daily metabolic equivalent of task (MET; *n* = 34,646; 50.93%), and had a high Health Literacy Scale (HLS) score (*n* = 35,503; 52.19%) or intermediate HLS (*n* = 23,130; 34.44%). The baseline characteristics of participants with dementia, AD, or PD can be found in Supplementary Table S4.

### 3.2 Relationships between blood concentrations of BCAAs and the risks of dementia, AD, and PD

Supplementary Table S5 illustrates the association between different blood concentration ranges of the three BCAAs and the adjusted risks of dementia, AD, and PD.

The association between blood concentrations of isoleucine, leucine and valine and the risk of dementia was found to be significant by model 3. More specifically, participants with higher blood concentrations of isoleucine, leucine and valine experienced a decreased risk of dementia, isoleucine with the greatest decrease observed in those whose concentrations were equal to or greater than the third quartile (HR: 0.82, 95% CI: 0.72–0.94, P for trend = 0.0066). Similarly, leucine with the largest decrease observed in those in the second quartile [HR: 0.72, 95% CI: 0.63–0.82, P for trend = 0.0001 (model 3)]. Furthermore, valine with the largest decrease observed in those in the second quartile in the third quartile in model 3 [HR: 0.77, 95% CI: 0.68–0.89, P for trend = 0.0004 (model 3)] (see Supplementary Table S5).

Blood concentrations of isoleucine, leucine and valine were significantly associated with the risk of AD in all models. Specifically, the risk of AD was lower in participants with different concentrations of isoleucine, with the largest decrease in risk observed in those with Q3 concentrations of isoleucine in model 3 (HR: 0.64, 95% CI: 0.49–0.86, P for trend = 0.0031). Similarly, with the greatest reductions in risk observed in those with Q2 concentrations of leucine, 35% decreases in risk in models 3 [HR: 0.65, 95% CI: 0.49–0.85, P for trend = 0.0093 (model 3)]. Moreover, the risk of AD was lower in participants with valine concentrations, with the greatest decrease in risk observed in those with Q3 concentrations of valine in model 3 [HR: 0.63, 95% CI: 0.47–0.84, P for trend = 0.0014 (model 3)] (see Supplementary Table S5).

**Figure 1:**
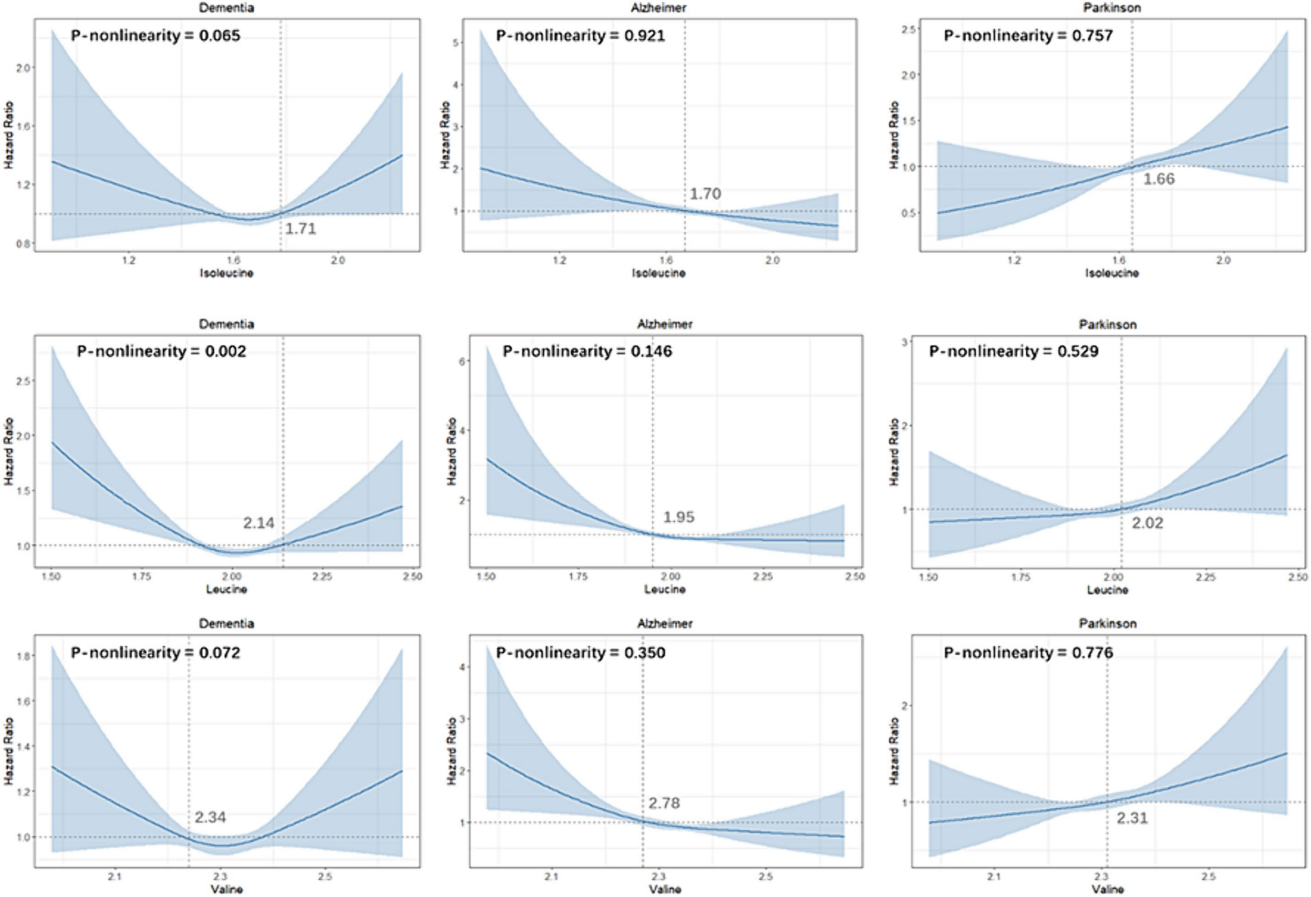
Dose-Response Association between BCAAs and Neurodegenerative Disorders.

A restricted cubic spline was employed to evaluate the dose–response association between BCAAs and three neurodegenerative disorders, illustrated in Figure 1. Our analysis revealed a nonlinear connection between leucine and the incidence of dementia (P-nonlinearity for leucine = 0.002). Specifically, a blood concentration of leucine at 2.14 mmoL/L was found to be associated with the lowest risk of dementia.

### 3.3 Interaction analysis of PRSs and HLS with dementia, AD, and PD

Based on the genetic risk score (PRS) and the Healthy Lifestyle Score (HLS), we classified the population into three levels: low, medium, and high. We then analyzed the impact of BCAA on dementia risk, AD, and PD in different PRS groups (see Supplementary Table S6) and HLS groups (see Supplementary Table S7).

In the high-risk genetic group, using the low-level valine as a reference, the high-level valine could reduce the risk of developing dementia and AD, and the HR for the risk of developing dementia and AD were 0.76 (95% CI: 0.61, 0.95), *p* = 0.0166, and 0.64 (95% CI: 0.43, 0.96), *p* = 0.0337, respectively. The interaction between different genetic risk and different BCAAs level does not show a significant impact on the risk of developing dementia and AD (see Supplementary Table S6).

In the healthy lifestyle group, high isoleucine and valine levels reduced the risk of dementia relative to low isoleucine and valine levels, with an HR of 0.65 (95% CI: 0.44, 0.96) for isoleucine, *p* = 0.0306; The HR for valine was 0.66 (95% CI: 0.45, 0.98), *p* = 0.0389; using the low valine level as a reference, the high valine level reduced the risk of AD with an HR of 0.37 (95% CI: 0.18, 0.79), *p* = 0.0093. The interaction between different healthy lifestyle and different BCAAs level does not show a significant impact on the risk of developing dementia and AD (see Supplementary Table S7).

### 3.4 Sensitivity analysis

A significant association between blood isoleucine, leucine and valine concentrations and the risk of developing dementia and AD was found by model 3. More specifically, participants with higher blood concentrations of isoleucine, leucine and valine experienced a decreased risk of dementia (Supplementary Table S8).

Supplementary Table S9 presents the stratified analyses, conducted based on various variables such as age, BMI, smoking status, alcohol consumption status and education level.

Supplementary Table S10 presents the association between various levels of Isoleucine, Leucine, and Valine and the presence or absence of APOEε4, with regards to dementia risk, AD risk, and PD risk. The results indicate that there is no interaction between these amino acids and the aforementioned risks. High leucine level was associated with a reduced risk of developing dementia in patients carrying APOEε4, HR 0. 77, 95% CI: 0.61–0.97, *p* = 0.0249, and high valine level was associated with a reduced risk of developing AD in patients not carrying APOEε4, HR 0. 41, 95% CI: 0.24–0.68, *p* = 0.0004. The interaction of whether to carry APOEε4 with different level of BCAAs on the risk of developing dementia and AD was not significant. 0.68, *p* = 0.0004. The interaction between the presence or absence of APOEε4 and the different level of BCAAs on the risk of dementia and AD development was not significant. Association between BCAA and Dementia, Alzheimer’s disease, Parkinson’s disease, excluding cases in the 2 years before follow-up was shown in Supplementary Table S11.

## 4 Discussion

This prospective cohort study involved 68,032 participants from the UK Biobank and aimed to assess the association between blood concentrations of BCAAs and the risks of dementia, AD, and PD. Our findings indicated that blood levels of BCAAs is associated with increased risk of dementia and AD.

BCAAs, as essential amino acids, have been shown to be strongly associated with disorders such as dementia, AD and mild cognitive impairment (MCI) (Jasbi et al., 2021). In a prospective study of eight cohorts, lower levels of BCAAs (e.g., valine) were associated with an increased risk of dementia and AD (Tynkkynen et al., 2018); Similarly, in a Mendelian randomization study, a causal association was found with lower levels of BCAAs in AD patients (Qian et al., 2023); In Alzheimer’s Disease Neuroimaging Initiative-1 (ADNI-1) cohort, serum valine levels were decreased in AD patients compared with patients with other diseases (Xiong et al., 2022). Another study using COX proportional regression modeling to explore the relationship between BCAAs and dementia risk (Zhang et al., 2022), also yielded similar results.

Nevertheless, there were elevated BCAA concentrations in the serum of AD patients in another sample study (Li et al., 2018). Similarly, serum isoleucine levels were elevated in dementia patients, compared with healthy controls (Socha et al., 2020). Combined with the results of this study, we speculate that these inconsistent results occurred in ordinary observational studies due to the limited sample size and dynamic changes in blood concentrations and degradation of BCAA (Lynch and Adams, 2014; Siddik et al., 2022).

Interestingly, a study examined the impact of dietary proteins/Amino LP7 (comprising leucine, phenylalanine, and lysine, along with isoleucine, histidine, valine, and tryptophan) on various components of the disease progression in rTg4510 mice, a mouse model of tauopathy, indicated that Amino LP7 supplementation led to improvements in tau-related brain atrophy, synaptic loss, and microglial activation (Sato et al., 2021). Another study investigated the impact of consuming Amino LP7 on cognitive function as the primary focus and psychosocial function as the secondary concern among middle-aged and elderly individuals. The results showed that a daily intake of 6 g of amino LP7 led to enhanced attention (Suzuki et al., 2020). The consumption of essential amino acids has been linked to preventing low protein levels, although the specific mechanism of this intervention and its long-term effects remain unclear. For older adults aiming to address both cognitive decline and frailty, promoting health through readily available methods like supplements could serve as a beneficial complementary.

The mTOR signaling axis has been shown to be severely deregulated in various neurodegenerative diseases, particularly AD (Lee et al., 2015). Postmortem examinations of AD patients’ brains have revealed hyperactivation of the PI3K-Akt–mTOR signaling pathway (Sun et al., 2014). Of note, mTORC1 activity is responsive to the bioavailability of amino acids, particularly BCAAs. Leucine, for instance, is a potent mTORC1 activator (Gran and Cameron-Smith, 2011). Of all three proteinogenic BCAAs, this amino acid possesses the strongest ability to activate the mTOR signaling pathway, valine induces mTOR activity, but its isoform does not (Polis and Samson, 2020). In a study of metabolic abnormalities in BCAAs and the development of AD, they found that leucine, but not valine and isoleucine, upregulates the phosphorylation of tau proteins in neurons isolated from AD mice via mTOR activation (Li et al., 2018). Therefore, further evidence is still needed to elucidate the physiological and pathological functions of branched-chain amino acids in different tissues and conditions.

Growing research indicates that AD is essentially a metabolic disorder, exhibiting molecular and biochemical features similar to diabetes and other insulin-resistant conditions. Consequently, some scholars have proposed the term “Type 3 diabetes” for AD, as it has been observed that high levels of BCAAs in plasma are associated with insulin resistance (Lackey et al., 2013; Badoud et al., 2014). When discussing the increased risk, one of the mechanisms is that AD is fundamentally a metabolic disease, and its molecular and biochemical characteristics correspond to diabetes and other peripheral insulin resistant diseases. High levels of plasma branched chain amino acids are associated with insulin resistance, therefore BCAAs may promote disease occurrence through insulin resistance.

Current research indicates that various factors, such as genetic predisposition, lifestyle choices, age, BMI, education level, smoking habit, and alcohol consumption, all contribute to an increased risk of developing dementia, AD, and PD (Livingston et al., 2020; Zhao et al., 2020). In view of this, we conducted sensitivity analyses to account for these covariates and mitigate their potential impact on the risks of dementia, AD, and PD. In this study, high isoleucine and valine level reduced the risk of dementia; high valine level reduced the risk of AD. This highlights the benefits of adherence to a healthy lifestyle for patients with neurodegenerative diseases.

Heritability in Alzheimer’s disease has been estimated to be in the range of 48–79% (Pedersen et al., 2004; Gatz et al., 2006). Studies have identified the apolipoprotein E (APOE) ε4 allele as significant loci for AD (Lambert et al., 2013; Escott-Price et al., 2014; Medland et al., 2014). One study found a significant association between AD PRSs and left hippocampal volume, with higher risk associated with lower left hippocampal volume. This effect remained when the APOE gene was excluded, suggesting that the relationship between hippocampal volume and AD is the result of multiple genetic factors and not exclusively variability in the APOE gene. This provides new insights into possible biological mechanisms of neurodegenerative processes. PRSs, which are based on the additive effect of multiple loci across the genome, may be better suited to capture the variance explained by common alleles (Dudbridge, 2013). PRSs based on the most recent GWASs have considerable predictive utility for AD risk (Escott-Price et al., 2015). Importantly, it has been suggested that genetic studies have demonstrated the high complexity of neurodegenerative traits, whose risk is modulated by a large number of variants, with either small effect or very low frequency in the population.

The present study suggests that BCAAs may be protective factors for dementia and AD. Notably, AD is the most common cause of dementia; therefore, the effects of BCAAs on dementia and AD are likely to be similar. Isoleucine, leucine and valine are essential amino acids in the human body, and their circulating levels are highly dependent on dietary intake, therefore, a decrease in the concentration of essential amino acids in the blood may indicate an underlying nutritional deficiency in preclinical dementia (Sato et al., 2021). Studies have shown that glutamate binds to cell surface receptors, the alpha-amino-3-hydroxy-5-methyl-4-isoxazole-propanoic acid receptor and the N-methyl-D- aspartate receptor. Dysfunction of the N-methyl-D-aspartate receptor causes glutamate overdose, leading to neuronal toxicity, and BCAAs may promote glutamate catabolism by activating glutamate dehydrogenase, which in turn helping to buffer toxic concentrations of glutamate (Yielding and Tomkins, 1961; Yudkoff, 2017; Polis and Samson, 2020). Thus, glutamate catabolism has the potential to mediate the relationship between blood BCAAs and the risk of developing dementia and AD. It has been found that the association between elevated hereditary isoleucine levels and AD may be mediated by attenuated brain serotonin levels and diminished serotonin signaling, which leads to the accumulation of amyloid plaque load, reduced neuronal survival and reduced occurrence of adult hippocampal neurogenesis (Larsson and Markus, 2017). In humans and mice, elevated serum levels of BCAAs have been associated with AD, and leucine-rich diets have been shown to activate phospho-Tau via a mammalian target of rapamycin (mTOR)-dependent mechanism, which significantly increased the phosphorylation levels of tau in the brain tissue of AD mice (Li et al., 2018).

However, our study differs from others in several aspects. First, in terms of the number of diseases, three neurodegenerative diseases were selected for analysis in this study; second, from a methodological point of view, this study was analyzed on the basis of the COX proportional regression model also using restricted cubic spline curves, polygenic risk scores, and sensitivity analyses, which provide an important guarantee of the reliability of the results. Unlike previous studies, we are the first to conclude that the risk of dementia is lowest when the concentration of leucine in the blood reaches 2.14 mmol/L. This provides a scientific basis for future research on the relationship between the dosage of BCAAs and the occurrence and development of neurodegenerative diseases.

There are several limitations to this study. Firstly, our analysis was confined to participants in UK Biobank, a notable constraint arises in the shape of sample bias, particularly, the UK Biobank predominantly comprises Caucasian individuals, making up around 88% of the total subjects (Fry et al., 2017). And considering that the BCAAs used are similar in time to the outcome data, there may be some bias in the results. The results of our study may not be generalizable to the broader diagnoses of disease, particularly dementia and AD. Nonetheless, valid assessments of exposure and disease based on UK Biobank data are widely accessible. Moreover, previous studies investigating the relationship between blood concentrations of BCAAs and the risks of dementia, AD, and PD have largely been cross-sectional, with limited cohort studies conducted. Additionally, our analysis strictly controlled for numerous covariates, and a restricted cubic spline was employed to assess the dose–response relationship between BCAAs and the risks of dementia, AD, and PD. Consequently, the findings of this study may serve as a foundation for identifying potential therapies and biomarkers for dementia, AD, and PD.

This study identified a negative association between blood concentrations of BCAAs and the risks of dementia and AD, suggesting that BCAAs could serve as biomarkers for these neurodegenerative diseases. On the other hand, the positive relationship between blood concentrations of BCAAs and the risk of PD may be influenced by other factors. Therefore, further investigation is needed to understand the mechanisms through which BCAAs impact the development of dementia, AD, and PD. It is imperative to explore these mechanisms in future studies in order to better comprehend the potential of intervening in BCAA metabolism as a preventive measure against these diseases.

## Data availability statement

The original contributions presented in the study are included in the article/supplementary material, further inquiries can be directed to the corresponding authors.

## Ethics statement

The studies involving humans were approved by the Research Ethics Committee (Reference 11/NW/0382). The studies were conducted in accordance with the local legislation and institutional requirements. The participants provided their written informed consent to participate in this study.

## Author contributions

YF: Conceptualization, Methodology, Writing – review & editing. HR: Data curation, Formal analysis, Writing – review & editing. XG: Writing – original draft, Writing – review & editing. LH: Writing – original draft, Writing – review & editing. YW: Formal analysis, Writing – original draft.

## References

[ref1] Badoud F. Lam K. P. DiBattista A. Perreault M. Zulyniak M. A. Cattrysse B.. (2014). Serum and adipose tissue amino acid homeostasis in the metabolically healthy obese. J. Proteome Res. 13, 3455–3466. doi: 10.1021/pr500416v, PMID: 24933025

[ref2] Chevli P. A. Freedman B. I. Hsu F. C. Xu J. Rudock M. E. Ma L.. (2021). Plasma metabolomic profiling in subclinical atherosclerosis: the diabetes heart study. Cardiovasc. Diabetol. 20:231. doi: 10.1186/s12933-021-01419-y, PMID: 34876126 PMC8653597

[ref3] Chouraki V. Reitz C. Maury F. Bis J. C. Bellenguez C. Yu L.. (2016). Evaluation of a genetic risk score to improve risk prediction for Alzheimer's disease. J. Alzheimers Dis. 53, 921–932. doi: 10.3233/jad-150749, PMID: 27340842 PMC5036102

[ref4] Dudbridge F. (2013). Power and predictive accuracy of polygenic risk scores. PLoS Genet. 9:e1003348. doi: 10.1371/journal.pgen.1003348, PMID: 23555274 PMC3605113

[ref5] Escott-Price V. Bellenguez C. Wang L. S. Choi S. H. Harold D. Jones L.. (2014). Gene-wide analysis detects two new susceptibility genes for Alzheimer's disease. PLoS One 9:e94661. doi: 10.1371/journal.pone.0094661, PMID: 24922517 PMC4055488

[ref6] Escott-Price V. Sims R. Bannister C. Harold D. Vronskaya M. Majounie E.. (2015). Common polygenic variation enhances risk prediction for Alzheimer's disease. Brain 138, 3673–3684. doi: 10.1093/brain/awv268, PMID: 26490334 PMC5006219

[ref7] Flores-Guerrero J. L. Groothof D. Connelly M. A. Otvos J. D. Bakker S. J. L. Dullaart R. P. F. (2019). Concentration of branched-chain amino acids is a strong risk marker for incident hypertension. Hypertension 74, 1428–1435. doi: 10.1161/hypertensionaha.119.13735, PMID: 31587574

[ref8] Fry A. Littlejohns T. J. Sudlow C. Doherty N. Adamska L. Sprosen T.. (2017). Comparison of sociodemographic and health-related characteristics of UK biobank participants with those of the general population. Am. J. Epidemiol. 186, 1026–1034. doi: 10.1093/aje/kwx246, PMID: 28641372 PMC5860371

[ref9] Gatz M. Reynolds C. A. Fratiglioni L. Johansson B. Mortimer J. A. Berg S.. (2006). Role of genes and environments for explaining Alzheimer disease. Arch. Gen. Psychiatry 63, 168–174. doi: 10.1001/archpsyc.63.2.168, PMID: 16461860

[ref10] GBD 2015 Neurological Disorders Collaborator Group (2017). Global, regional, and national burden of neurological disorders during 1990-2015: a systematic analysis for the global burden of disease study 2015. Lancet Neurol. 16, 877–897. doi: 10.1016/s1474-4422(17)30299-5, PMID: 28931491 PMC5641502

[ref11] Grajeda-Iglesias C. Aviram M. (2018). Specific amino acids affect cardiovascular diseases and atherogenesis via protection against macrophage foam cell formation. Rambam Maimonides Med. J. 9:e0022. doi: 10.5041/RMMJ.10337 29944113 PMC6115485

[ref12] Gran P. Cameron-Smith D. (2011). The actions of exogenous leucine on mTOR signalling and amino acid transporters in human myotubes. BMC Physiol. 11:10. doi: 10.1186/1472-6793-11-10, PMID: 21702994 PMC3141572

[ref13] Hanagasi H. A. Tufekcioglu Z. Emre M. (2017). Dementia in Parkinson's disease. J. Neurol. Sci. 374, 26–31. doi: 10.1016/j.jns.2017.01.012 28088312

[ref14] James B. D. Bennett D. A. (2019). Causes and patterns of dementia: an update in the era of redefining Alzheimer's disease. Annu. Rev. Public Health 40, 65–84. doi: 10.1146/annurev-publhealth-040218-043758, PMID: 30642228

[ref15] Jasbi P. Shi X. Chu P. Elliott N. Hudson H. Jones D.. (2021). Metabolic profiling of neocortical tissue discriminates Alzheimer's disease from mild cognitive impairment, high pathology controls, and Normal controls. J. Proteome Res. 20, 4303–4317. doi: 10.1021/acs.jproteome.1c00290, PMID: 34355917 PMC11060066

[ref16] Lackey D. E. Lynch C. J. Olson K. C. Mostaedi R. Ali M. Smith W. H.. (2013). Regulation of adipose branched-chain amino acid catabolism enzyme expression and cross-adipose amino acid flux in human obesity. Am. J. Physiol. Endocrinol. Metab. 304, E1175–E1187. doi: 10.1152/ajpendo.00630.2012, PMID: 23512805 PMC3680678

[ref17] Lambert J. C. Ibrahim-Verbaas C. A. Harold D. Naj A. C. Sims R. Bellenguez C.. (2013). Meta-analysis of 74,046 individuals identifies 11 new susceptibility loci for Alzheimer's disease. Nat. Genet. 45, 1452–1458. doi: 10.1038/ng.2802, PMID: 24162737 PMC3896259

[ref18] Larsson S. C. Markus H. S. (2017). Branched-chain amino acids and Alzheimer’s disease: a mendelian randomization analysis. Sci. Rep. 7:13604. doi: 10.1038/s41598-017-12931-1 29051501 PMC5648806

[ref19] Lee J. H. Tecedor L. Chen Y. H. Monteys A. M. Sowada M. J. Thompson L. M.. (2015). Reinstating aberrant mTORC1 activity in Huntington's disease mice improves disease phenotypes. Neuron 85, 303–315. doi: 10.1016/j.neuron.2014.12.019, PMID: 25556834 PMC4355620

[ref20] Li H. Ye D. Xie W. Hua F. Yang Y. Wu J.. (2018). Defect of branched-chain amino acid metabolism promotes the development of Alzheimer's disease by targeting the mTOR signaling. Biosci. Rep. 38:BSR20180127. doi: 10.1042/BSR20180127 29802157 PMC6028749

[ref21] Lim L. L. Lau E. S. H. Fung E. Lee H. M. Ma R. C. W. Tam C. H. T.. (2020). Circulating branched-chain amino acids and incident heart failure in type 2 diabetes: the Hong Kong diabetes register. Diabetes Metab. Res. Rev. 36:e3253. doi: 10.1002/dmrr.3253, PMID: 31957226

[ref22] Livingston G. Huntley J. Sommerlad A. Ames D. Ballard C. Banerjee S.. (2020). Dementia prevention, intervention, and care: 2020 report of the lancet commission. Lancet 396, 413–446. doi: 10.1016/s0140-6736(20)30367-6, PMID: 32738937 PMC7392084

[ref23] Lynch C. J. Adams S. H. (2014). Branched-chain amino acids in metabolic signalling and insulin resistance. Nat. Rev. Endocrinol. 10, 723–736. doi: 10.1038/nrendo.2014.171, PMID: 25287287 PMC4424797

[ref24] Medland S. E. Jahanshad N. Neale B. M. Thompson P. M. (2014). Whole-genome analyses of whole-brain data: working within an expanded search space. Nat. Neurosci. 17, 791–800. doi: 10.1038/nn.3718, PMID: 24866045 PMC4300949

[ref25] Nie C. He T. Zhang W. Zhang G. Ma X. (2018). Branched chain amino acids: beyond nutrition metabolism. Int. J. Mol. Sci. 19:954. doi: 10.3390/ijms19040954 29570613 PMC5979320

[ref26] Pedersen N. L. Gatz M. Berg S. Johansson B. (2004). How heritable is Alzheimer's disease late in life? Findings from Swedish twins. Ann. Neurol. 55, 180–185. doi: 10.1002/ana.10999, PMID: 14755721

[ref27] Polis B. Samson A. (2020). Role of the metabolism of branched-chain amino acids in the development of Alzheimer's disease and other metabolic disorders. Neural Regen. Res. 15, 1460–1470. doi: 10.4103/1673-5374.274328 31997805 PMC7059578

[ref28] Qian X.-H. Liu X.-L. Zhang B. Lin Y. Xu J. H. Ding G. Y.. (2023). Investigating the causal association between branched-chain amino acids and Alzheimer's disease: a bidirectional mendelian randomized study. Front. Nutr. 10:1103303. doi: 10.3389/fnut.2023.1103303 37063328 PMC10102518

[ref29] Sato H. Takado Y. Toyoda S. Tsukamoto-Yasui M. Minatohara K. Takuwa H.. (2021). Neurodegenerative processes accelerated by protein malnutrition and decelerated by essential amino acids in a tauopathy mouse model. Sci. Adv. 7:eabd5046. doi: 10.1126/sciadv.abd5046, PMID: 34678069 PMC8535828

[ref30] Siddik M. A. B. Mullins C. A. Kramer A. Shah H. Gannaban R. B. Zabet-Moghaddam M.. (2022). Branched-chain amino acids are linked with Alzheimer's disease-related pathology and cognitive deficits. Cells 11:3523. doi: 10.3390/cells11213523 36359919 PMC9658564

[ref31] Socha E. Kośliński P. Koba M. Mądra-Gackowska K. Kędziora-Kornatowska K. Gackowski M.. (2020). Amino acid levels as potential biomarker of elderly patients with dementia. Brain Sci. 10:914. doi: 10.3390/brainsci10120914 33260889 PMC7760342

[ref32] Sudlow C. Gallacher J. Allen N. Beral V. Burton P. Danesh J.. (2015). UK biobank: an open access resource for identifying the causes of a wide range of complex diseases of middle and old age. PLoS Med. 12:e1001779. doi: 10.1371/journal.pmed.1001779, PMID: 25826379 PMC4380465

[ref33] Sun Y. X. Ji X. Mao X. Xie L. Jia J. Galvan V.. (2014). Differential activation of mTOR complex 1 signaling in human brain with mild to severe Alzheimer's disease. J. Alzheimers Dis. 38, 437–444. doi: 10.3233/jad-131124, PMID: 23979023

[ref34] Suzuki H. Yamashiro D. Ogawa S. Kobayashi M. Cho D. Iizuka A.. (2020). Intake of seven essential amino acids improves cognitive function and psychological and social function in middle-aged and older adults: a double-blind, randomized, placebo-controlled trial. Front. Nutr. 7:586166. doi: 10.3389/fnut.2020.586166, PMID: 33324669 PMC7724102

[ref35] Tynkkynen J. Chouraki V. van der Lee S. J. Hernesniemi J. Yang Q. Li S.. (2018). Association of branched-chain amino acids and other circulating metabolites with risk of incident dementia and Alzheimer's disease: a prospective study in eight cohorts. Alzheimers Dement. 14, 723–733. doi: 10.1016/j.jalz.2018.01.003, PMID: 29519576 PMC6082422

[ref36] Wang X. Sun G. Feng T. Zhang J. Huang X. Wang T.. (2019). Sodium oligomannate therapeutically remodels gut microbiota and suppresses gut bacterial amino acids-shaped neuroinflammation to inhibit Alzheimer's disease progression. Cell Res. 29, 787–803. doi: 10.1038/s41422-019-0216-x, PMID: 31488882 PMC6796854

[ref37] Xiong Y. L. Therriault J. Ren S. J. Jing X. J. Zhang H. The Alzheimer’s Disease Neuroimaging Initiative (2022). The associations of serum valine with mild cognitive impairment and Alzheimer's disease. Aging Clin. Exp. Res. 34, 1807–1817. doi: 10.1007/s40520-022-02120-0, PMID: 35362856

[ref38] Yan Z. Yang F. Wen S. Ding W. Si Y. Li R.. (2022). Longitudinal metabolomics profiling of serum amino acids in rotenone-induced Parkinson's mouse model. Amino Acids 54, 111–121. doi: 10.1007/s00726-021-03117-1, PMID: 35028704

[ref39] Yielding K. L. Tomkins G. M. (1961). An effect of L-leucine and other essential amino acids on the structure and activity of glutamic dehydrogenase. Proc. Natl. Acad. Sci. USA 47, 983–989. doi: 10.1073/pnas.47.7.983, PMID: 13787322 PMC221313

[ref40] Yoo H. S. Shanmugalingam U. Smith P. D. (2022). Potential roles of branched-chain amino acids in neurodegeneration. Nutrition 103–104:111762. doi: 10.1016/j.nut.2022.111762, PMID: 35843039

[ref41] Yudkoff M. (2017). Interactions in the metabolism of glutamate and the branched-chain amino acids and ketoacids in the CNS. Neurochem. Res. 42, 10–18. doi: 10.1007/s11064-016-2057-z, PMID: 27696119 PMC5285401

[ref42] Zhang X. Hu W. Wang Y. Wang W. Liao H. Zhang X.. (2022). Plasma metabolomic profiles of dementia: a prospective study of 110,655 participants in the UK biobank. BMC Med. 20:252. doi: 10.1186/s12916-022-02449-3 35965319 PMC9377110

[ref43] Zhao N. Ren Y. Yamazaki Y. Qiao W. Li F. Felton L. M.. (2020). Alzheimer's risk factors age, APOE genotype, and sex drive distinct molecular pathways. Neuron 106, 727–742.e6. doi: 10.1016/j.neuron.2020.02.034, PMID: 32199103 PMC7388065

