# Supplementary material for: Branched-chain amino acids and the risks of dementia, Alzheimer’s disease, and Parkinson’s disease
Source: Front Aging Neurosci. 2024 Apr 10;16:1369493. doi: 10.3389/fnagi.2024.1369493 (PMC11040674; doi:10.3389/fnagi.2024.1369493)
Supplement: Supplementary file 1 [file Data_Sheet_1.docx]

Supplementary Material

# Supplementary Figures and Tables

## Supplementary Figures


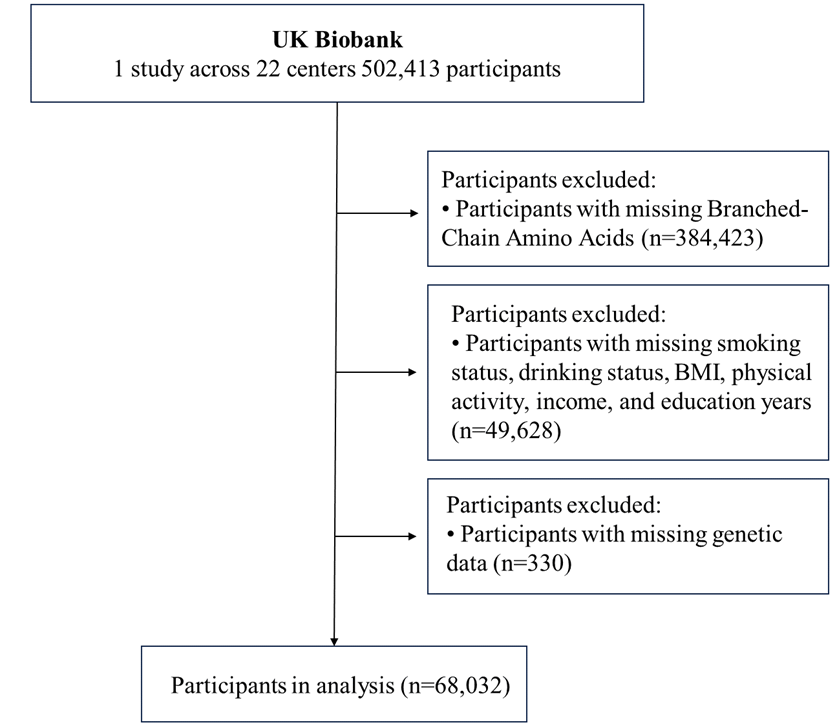


**Supplementary Figure 1.** Flow chart.

## Supplementary Tables

**Supplementary Table 1.** ICD-10 Codes for Neurodegenerative Diseases.

| Neurodegenerative Diseases | ICD-10 |
| --- | --- |
| Alzheimer's disease | G30 |
| Parkinson's disease | G20, G21, G22 |
| Dementia | F00, F01, F02, F03, F04 |

**Supplementary Table 2.** Descriptive statistics of the BCAA data.

| BCAA, mmol/l | N | Mean ± SD | Minimum | Maximum |
| --- | --- | --- | --- | --- |
| Isoleucine | 68032 | 1.675 ± 0.144 | -0.014 | 2.300 |
| leucine | 68032 | 1.991 ± 0.115 | 1.409 | 2.651 |
| Valine | 68032 | 2.300 ± 0.087 | 1.873 | 2.928 |

**Supplementary Table 3.** Description of lifestyle factors in the UK Biobank.

| Lifestyle factor | Questionnaire | Self-reported UK  Biobank field code | Healthy | Unhealthy |
| --- | --- | --- | --- | --- |
| Smoking status | "Do you smoke tobacco now?" and "In the past, how often have you smoked tobacco?" | 20116 | Past or never smoker | Current |
| Drinking status | “About how often do you drink alcohol?” and “Did you previously drink alcohol?” | 20117 | Never/previous | Current |
| Physical activity | IPAQ short form2– total time walking or moderate and vigorous-intensity physical activity (PA) in previous week | 22032 | ≥150 min/week moderate or ≥ 75 min/week vigorous PA | <150 min/week moderate or < 75 min/week vigorous PA |
| Dietary characteristics | Individual dietary components contributed directly to lifestyle score. |  |  |  |
| Fruit and vegetable intake | "About how many of …. would you eat per day?” Separate questions for pieces of fresh and dried fruit, tablespoons of salAlzheimer's Disease or cooked/raw vegetables. | 1309, 1319, 1289, 1299 | ≥ 6 portion/day | <6 portion/day |
| Red meat intake | "How often do you eat…?” Separate questions for Beef / lamb or mutton / pork (excluding processed meats such as ham or bacon). | 1369, 1379, 1389 | <7 portion/week | ≥7 portion/week |
| Processed meat intake | "How often do you eat processed meats (such as bacon, ham, sausages, meat pies, kebabs, burgers, chicken nuggets)?" | 1349 | <4 portion/week | ≥4 portion/week |

**Supplementary Table 4.** Characteristics of study population at baseline investigation.

| Characteristics | Total | Non-Dementia | Dementia | Non-Alzheimer’s disease | Alzheimer’s disease | Non-Parkinson’s disease | Parkinson’s disease |
| --- | --- | --- | --- | --- | --- | --- | --- |
| Age, y, mean ± SD | 56.51±8.13 | 56.36±8.10 | 64.06±5.46 | 56.47±8.12 | 65.02±4.38 | 56.46±8.13 | 63.08±5.02 |
| Sex, n (%) |  |  |  |  |  |  |  |
| Female | 34197(50.27) | 33701(50.52) | 496(37.29) | 34054(50.29) | 143(44.69) | 34066(50.42) | 131(27.99) |
| Male | 33835(49.73) | 33001(49.48) | 834(62.71) | 33658(49.71) | 177(55.31) | 33498(49.58) | 337(72.01) |
| Ethnicity, n (%) |  |  |  |  |  |  |  |
| Non-White | 3253(4.78) | 3209(4.81) | 44(3.31) | 3242(4.79) | 11(3.44) | 3236(4.79) | 17(3.63) |
| White | 64779(95.22) | 63493(95.19) | 1286(96.69) | 64470(95.21) | 309(96.56) | 64328(95.21) | 451(96.37) |
| [Education](javascript:;) [years](javascript:;), n (%) |  |  |  |  |  |  |  |
| ≤7 years | 8949(13.15) | 8563(12.84) | 386(29.02) | 8853(13.07) | 96(30.00) | 8855(13.11) | 94(20.09) |
| 7-10 years | 11057(16.25) | 10864(16.29) | 193(14.51) | 11006(16.25) | 51(15.94) | 10988(16.26) | 69(14.74) |
| 10-15 years | 12182(17.91) | 11945(17.91) | 237(17.82) | 12128(17.91) | 54(16.88) | 12103(17.91) | 79(16.88) |
| 15-20 years | 35844(52.69) | 35330(52.97) | 514(38.65) | 35725(52.76) | 119(37.19) | 35618(52.72) | 226(48.29) |
| BMI, kg/m2, n (%) |  |  |  |  |  |  |  |
| <25 | 22807(33.52) | 22425(33.62) | 382(28.72) | 22699(33.52) | 108(33.75) | 22669(33.55) | 138(29.49) |
| 25-30 | 29402(43.22) | 28836(43.23) | 566(42.56) | 29263(43.22) | 139(43.44) | 29179(43.19) | 223(47.65) |
| >30 | 15823(23.26) | 15441(23.15) | 382(28.72) | 15750(23.26) | 73(22.81) | 15716(23.26) | 107(22.86) |
| Smoking, n (%) |  |  |  |  |  |  |  |
| Never | 36877(54.21) | 36305(54.43) | 572(43.01) | 36716(54.22) | 161(50.31) | 36647(54.24) | 230(49.15) |
| Previous | 24102(35.43) | 23512(35.25) | 590(44.36) | 23966(35.39) | 136(42.50) | 23906(35.38) | 196(41.88) |
| Current | 7053(10.37) | 6885(10.32) | 168(12.63) | 7030(10.38) | 23(7.19) | 7011(10.38) | 42(8.97) |
| Drinking, n (%) |  |  |  |  |  |  |  |
| Nondrinker | 2344(3.45) | 2269(3.40) | 75(5.64) | 2323(3.43) | 21(6.56) | 2327(3.44) | 17(3.63) |
| Previous | 2234(3.28) | 2147(3.22) | 87(6.54) | 2218(3.28) | 16(5.00) | 2216(3.28) | 18(3.85) |
| Current | 63454(93.27) | 62286(93.38) | 1168(87.82) | 63171(93.29) | 283(88.44) | 63021(93.28) | 433(92.52) |
| MET, n (%) |  |  |  |  |  |  |  |
| <600 | 12792(18.80) | 12489(18.72) | 303(22.78) | 12727(18.80) | 65(20.31) | 12714(18.82) | 78(16.67) |
| 600-3000 | 34646(50.93) | 34009(50.99) | 637(47.89) | 34489(50.93) | 157(49.06) | 34384(50.89) | 262(55.98) |
| >3000 | 20594(30.27) | 20204(30.29) | 390(29.32) | 20496(30.27) | 98(30.63) | 20466(30.29) | 128(27.35) |
| HLS, n (%) |  |  |  |  |  |  |  |
| 1 | 25344(37.25) | 24898(37.33) | 446(33.53) | 25258(37.30) | 86(26.88) | 25186(37.28) | 158(33.76) |
| 2 | 35503(52.19) | 34821(52.20) | 682(51.28) | 35324(52.17) | 179(55.94) | 35248(52.17) | 255(54.49) |
| 3 | 7185(10.56) | 6983(10.47) | 202(15.19) | 7130(10.53) | 55(17.19) | 7130(10.55) | 55(11.75) |
| PRS, n (%) |  |  |  |  |  |  |  |
| Low | 22451(33.00) | 22142(33.20) | 309(23.23) | 22409(33.09) | 42(13.13) | 22311(33.02) | 140(29.91) |
| Intermediate | 23130(34.00) | 22749(34.11) | 381(28.65) | 23069(34.07) | 61(19.06) | 22967(33.99) | 163(34.83) |
| High | 22451(33.00) | 21811(32.70) | 640(48.12) | 22234(32.84) | 217(67.81) | 22286(32.99) | 165(35.26) |
| BCAAs, mmol/l  (mean ± SD) |  |  |  |  |  |  |  |
| Isoleucine | 1.68±0.15 | 1.67±0.14 | 1.68±0.15 | 1.67±0.12 | 1.65±0.15 | 1.67±0.14 | 1.69±0.14 |
| Leucine | 1.99±0.11 | 1.99±0.11 | 1.98±0.12 | 1.99±0.11 | 1.97±0.12 | 1.99±0.11 | 2.00±0.12 |
| Valine | 2.30±0.09 | 2.30±0.08 | 2.30±0.09 | 2.30±0.08 | 2.28±0.09 | 2.30±0.09 | 2.31±0.09 |

**Supplementary Table 5.** Association of branched-chain amino acids (BCAAs) with Dementia, Alzheimer's disease, and Parkinson's disease.

| BCAAs | No. Dementia cases/person-years | Dementia HR (95% CI) | | | No. Alzheimer’s Disease cases/ person-years | Alzheimer’s Disease HR (95% CI) | | | No. Parkinson’s Disease cases/ person-years | Parkinson’s Disease HR (95% CI) | | |
| --- | --- | --- | --- | --- | --- | --- | --- | --- | --- | --- | --- | --- |
|  |  | Model 1 | Model 2 | Model 3 |  | Model 1 | Model 2 | Model 3 |  | Model 1 | Model 2 | Model 3 |
| Isoleucine |  |  |  |  |  |  |  |  |  |  |  |  |
| Q1 | 439/281108 | Reference | Reference | Reference | 119/281869 | Reference | Reference | Reference | 132/281649 | Reference | Reference | Reference |
| Q2 | 428/280484 | 0.98 (0.86-1.12) | 0.87(0.76-0.99) | 0.84 (0.73-0.96) | 117/281228 | 0.99 (0.76-1.27) | 0.92 (0.71-1.19) | 0.92 (0.71-1.19) | 153/280894 | 1.16 (0.92-1.47) | 0.97 (0.77-1.22) | 0.99 (0.78-1.25) |
| Q3 | 463/290024 | 1.02 (0.89-1.16) | 0.9 (0.79-1.03) | 0.82 (0.72-0.94) | 84/290900 | 0.68 (0.52-0.9) | 0.65 (0.49-0.86) | 0.64 (0.48-0.86) | 183/290440 | 1.34 (1.07-1.68) | 1.08 (0.86-1.36) | 1.09 (0.87-1.38) |
| P for trend |  | 0.7687 | 0.1416 | 0.0066 |  | 0.0083 | 0.0030 | 0.0031 |  | 0.0093 | 0.4596 | 0.4202 |
| Leucine |  |  |  |  |  |  |  |  |  |  |  |  |
| Q1 | 477/280062 | Reference | Reference | Reference | 133/280872 | Reference | Reference | Reference | 135/280612 | Reference | Reference | Reference |
| Q2 | 393/281216 | 0.82 (0.72-0.94) | 0.74(0.64-0.84) | 0.72 (0.63-0.82) | 89/281951 | 0.66 (0.51-0.87) | 0.64 (0.49-0.84) | 0.65(0.49-0.85) | 145/281677 | 1.07 (0.85-1.35) | 0.89 (0.7-1.13) | 0.91 (0.72-1.16) |
| Q3 | 460/290338 | 0.92 (0.81-1.05) | 0.82(0.72-0.94) | 0.75 (0.66-0.86) | 98/291174 | 0.71 (0.54-0.92) | 0.7 (0.53-0.91) | 0.7 (0.53-0.92) | 188/290694 | 1.34 (1.08-1.67) | 1.05 (0.83-1.32) | 1.07 (0.84-1.35) |
| P for trend |  | 0.2324 | 0.0044 | 0.0001 |  | 0.0071 | 0.0074 | 0.0093 |  | 0.0077 | 0.5820 | 0.5027 |
| Valine |  |  |  |  |  |  |  |  |  |  |  |  |
| Q1 | 443/280353 | Reference | Reference | Reference | 125/281065 | Reference | Reference | Reference | 133/280821 | Reference | Reference | Reference |
| Q2 | 413/281203 | 0.93 (0.81-1.06) | 0.8 (0.7-0.92) | 0.77 (0.68-0.89) | 104/281947 | 0.83 (0.64-1.07) | 0.74 (0.57-0.97) | 0.76 (0.58-0.99) | 162/281603 | 1.21 (0.97-1.53) | 0.99 (0.79-1.25) | 1.02 (0.81-1.29) |
| Q3 | 474/290060 | 1.03 (0.9-1.17) | 0.87(0.76-0.99) | 0.77 (0.68-0.89) | 91/290985 | 0.7 (0.53-0.92) | 0.63 (0.48-0.83) | 0.63 (0.47-0.84) | 173/290559 | 1.26 (1-1.57) | 0.97 (0.77-1.22) | 0.98 (0.77-1.25) |
| P for trend |  | 0.6509 | 0.0442 | 0.0004 |  | 0.0090 | 0.0010 | 0.0014 |  | 0.0521 | 0.7570 | 0.8650 |

Model 1 was adjusted for age, sex, ethnicity, income, work, education, drink, smoke, BMI, SBP, hypertension, diabetes;

Model 2 was adjusted for age, sex, ethnicity, income, work, education, drink, smoke, BMI, SBP, hypertension, diabetes, stroke;

Model 3 was adjusted for age, sex, ethnicity, income, work, education, drink, smoke, BMI, SBP, hypertension, diabetes, stroke, HDL, LDL, TG, TC.

P value for trend calculated treating the BCAAs (tertiles) as a continuous variable.

**Supplementary Table 6.** Risk of incident Dementia, Alzheimer's disease, and Parkinson's disease stratified by Polygenic risk scores in model 3

| Disease | BCAAs | Polygenic risk scores (PRS) | | | | | | | | | P for Interaction |
| --- | --- | --- | --- | --- | --- | --- | --- | --- | --- | --- | --- |
|  |  | Low | | | Intermediate | | | High | | |  |
|  |  | Cases/  person-years | HR (95% CI) | P for trend | Cases/  person-years | HR (95% CI) | P for trend | Cases/  person-years | HR (95% CI) | P for trend |  |
| Dementia | Isoleucine |  |  |  |  |  |  |  |  |  | 0.83 |
|  | Low | 100/92732 | Reference | 0.132 | 125/95790 | Reference | 0.1477 | 212/92625 | Reference | 0.1945 |  |
|  | Intermediate | 112/92743 | 1.07 (0.79-1.45) |  | 105/95451 | 0.72 (0.54-0.95) |  | 210/92282 | 0.90 (0.73-1.12) |  |  |
|  | High | 97/95909 | 0.79 (0.57-1.09) |  | 151/98707 | 0.80 (0.61-1.06) |  | 218/95379 | 0.86 (0.69-1.08) |  |  |
|  | Leucine |  |  |  |  |  |  |  |  |  |  |
|  | Low | 118/92451 | Reference | 0.0029 | 128/95490 | Reference | 0.0798 | 228/92120 | Reference | 0.0562 | 0.7613 |
|  | Intermediate | 92/92806 | 0.70 (0.52-0.96) |  | 110/95679 | 0.71 (0.53-0.94) |  | 192/92750 | 0.73 (0.59-0.91) |  |  |
|  | High | 99/96127 | 0.62 (0.45-0.85) |  | 143/98779 | 0.77 (0.58-1.02) |  | 220/95414 | 0.80 (0.65-1.00) |  |  |
|  | Valine |  |  |  |  |  |  |  |  |  |  |
|  | Low | 107/92538 | Reference | 0.042 | 122/95626 | Reference | 0.0721 | 214/92223 | Reference | 0.0166 | 0.7508 |
|  | Intermediate | 94/92730 | 0.72 (0.53-0.99) |  | 113/95678 | 0.77 (0.58-1.02) |  | 209/92705 | 0.86 (0.69-1.06) |  |  |
|  | High | 108/96116 | 0.72 (0.52-0.98) |  | 146/98643 | 0.76 (0.57-1.01) |  | 217/95357 | 0.76 (0.61-0.95) |  |  |
| Alzheimer's Disease | Isoleucine |  |  |  |  |  |  |  |  |  | 0.9776 |
|  | Low | 15/92955 | Reference | 0.2249 | 22/96054 | Reference | 0.0704 | 80/92894 | Reference | 0.0634 |  |
|  | Intermediate | 17/92991 | 0.98 (0.46-2.10) |  | 18/95638 | 0.68 (0.35-1.33) |  | 83/92576 | 0.99 (0.70-1.39) |  |  |
|  | High | 10/96113 | 0.58 (0.24-1.39) |  | 21/99011 | 0.52 (0.26-1.06) |  | 54/95765 | 0.68 (0.46-1.01) |  |  |
|  | Leucine |  |  |  |  |  |  |  |  |  | 0.7193 |
|  | Low | 16/92702 | Reference | 0.2476 | 24/95745 | Reference | 0.0436 | 91/92419 | Reference | 0.0555 |  |
|  | Intermediate | 13/93036 | 0.85 (0.39-1.84) |  | 14/95886 | 0.48 (0.24-0.97) |  | 63/93041 | 0.65 (0.46-0.94) |  |  |
|  | High | 13/96321 | 0.60 (0.26-1.42) |  | 23/99072 | 0.49 (0.25-0.98) |  | 63/95775 | 0.71 (0.49-1.03) |  |  |
|  | Valine |  |  |  |  |  |  |  |  |  | 0.1752 |
|  | Low | 18/92710 | Reference | 0.096 | 23/95880 | Reference | 0.0707 | 83/92518 | Reference | 0.0337 |  |
|  | Intermediate | 11/93000 | 0.52 (0.23-1.16) |  | 16/95861 | 0.52 (0.26-1.05) |  | 78/92994 | 0.92 (0.66-1.30) |  |  |
|  | High | 13/96349 | 0.51 (0.23-1.14) |  | 23/99072 | 0.52 (0.26-1.04) |  | 56/95722 | 0.64 (0.43-0.96) |  |  |
| Parkinson's Disease | Isoleucine |  |  |  |  |  |  |  |  |  | 0.6588 |
|  | Low | 38/92796 | Reference | 0.6032 | 42/95948 | Reference | 0.4619 | 51/92936 | Reference | 0.7006 |  |
|  | Intermediate | 45/92811 | 1.04 (0.65-1.66) |  | 55/95503 | 1.19 (0.77-1.84) |  | 54/92557 | 0.89 (0.59-1.35) |  |  |
|  | High | 57/95957 | 1.13 (0.71-1.79) |  | 66/98777 | 1.19 (0.77-1.85) |  | 60/95698 | 0.92 (0.61-1.38) |  |  |
|  | Leucine |  |  |  |  |  |  |  |  |  | 0.7609 |
|  | Low | 40/92507 | Reference | 0.5896 | 43/95610 | Reference | 0.9374 | 51/92493 | Reference | 0.8503 |  |
|  | Intermediate | 43/92915 | 0.97 (0.60-1.56) |  | 57/95769 | 1.04 (0.68-1.61) |  | 47/92996 | 0.72 (0.47-1.12) |  |  |
|  | High | 57/96142 | 1.12 (0.71-1.79) |  | 63/98850 | 1.02 (0.66-1.59) |  | 67/95702 | 1.00 (0.66-1.51) |  |  |
|  | Valine |  |  |  |  |  |  |  |  |  | 0.9436 |
|  | Low | 39/92528 | Reference | 0.5377 | 45/95764 | Reference | 0.2728 | 48/92572 | Reference | 0.8636 |  |
|  | Intermediate | 43/92851 | 0.92 (0.57-1.49) |  | 65/95669 | 1.27 (0.84-1.91) |  | 56/92989 | 0.87 (0.57-1.33) |  |  |
|  | High | 58/96185 | 1.14 (0.71-1.80) |  | 53/98796 | 0.79 (0.49-1.25) |  | 61/95630 | 0.95 (0.63-1.45) |  |  |

Model 3 was adjusted for age, sex, ethnicity, income, work, education, drink, smoke, BMI, SBP, hypertension, diabetes, stroke, HDL, LDL, TG, TC.

**Supplementary Table 7.** Risk of incident Dementia, Alzheimer's disease, and Parkinson's disease stratified by Healthy lifestyle scores (HLS) in model 3.

| Disease | BCAAs | Healthy lifestyle scores (HLS) | | | | | | | | | P for Interaction |
| --- | --- | --- | --- | --- | --- | --- | --- | --- | --- | --- | --- |
|  |  | Low | | | Intermediate | | | High | | |  |
|  |  | Cases/  person-years | HR (95% CI) | P for trend | Cases/  person-years | HR (95% CI) | P for trend | Cases/  person-years | HR (95% CI) | P for trend |  |
| Dementia | Isoleucine |  |  |  |  |  |  |  |  |  | 0.4615 |
|  | Low | 143/104523 | Reference | 0.4997 | 218/147320 | Reference | 0.1303 | 72/29292 | Reference | 0.0306 |  |
|  | Intermediate | 152/104176 | 0.96 (0.74-1.25) |  | 213/147064 | 0.88 (0.71-1.08) |  | 72/29255 | 0.88 (0.61-1.27) |  |  |
|  | High | 151/108074 | 0.91 (0.70-1.19) |  | 251/151449 | 0.85 (0.69-1.05) |  | 58/30463 | 0.65 (0.44-0.96) |  |  |
|  | Leucine |  |  |  |  |  |  |  |  |  | 0.5277 |
|  | Low | 172/104003 | Reference | 0.0073 | 229/146927 | Reference | 0.0292 | 71/29325 | Reference | 0.0751 |  |
|  | Intermediate | 129/104555 | 0.69 (0.53-0.89) |  | 199/147387 | 0.72 (0.58-0.89) |  | 67/29187 | 0.97 (0.67-1.40) |  |  |
|  | High | 145/108214 | 0.70 (0.54-0.90) |  | 254/151519 | 0.78 (0.63-0.97) |  | 64/30499 | 0.70 (0.48-1.04) |  |  |
|  | Valine |  |  |  |  |  |  |  |  |  | 0.6689 |
|  | Low | 145/104140 | Reference | 0.3983 | 225/146998 | Reference | 0.0016 | 71/29289 | Reference | 0.0389 |  |
|  | Intermediate | 143/104474 | 0.84 (0.65-1.10) |  | 210/147402 | 0.79 (0.64-0.97) |  | 64/29305 | 0.80 (0.55-1.16) |  |  |
|  | High | 158/108158 | 0.89 (0.68-1.16) |  | 247/151433 | 0.71 (0.57-0.87) |  | 67/30417 | 0.66 (0.45-0.98) |  |  |
| Alzheimer's Disease | Isoleucine |  |  |  |  |  |  |  |  |  | 0.545 |
|  | Low | 31/104762 | Reference | 0.2072 | 67/147703 | Reference | 0.0205 | 18/29406 | Reference | 0.121 |  |
|  | Intermediate | 34/104450 | 1.02 (0.58-1.77) |  | 61/147462 | 0.84 (0.57-1.23) |  | 26/29352 | 1.10 (0.58-2.10) |  |  |
|  | High | 21/108361 | 0.66 (0.35-1.25) |  | 51/151912 | 0.60 (0.40-0.92) |  | 11/30588 | 0.54 (0.25-1.17) |  |  |
|  | Leucine |  |  |  |  |  |  |  |  |  | 0.6206 |
|  | Low | 39/104291 | Reference | 0.0293 | 73/147336 | Reference | 0.0408 | 20/29420 | Reference | 0.1475 |  |
|  | Intermediate | 25/104792 | 0.60 (0.34-1.06) |  | 48/147767 | 0.59 (0.39-0.88) |  | 20/29300 | 1.06 (0.55-2.04) |  |  |
|  | High | 22/108490 | 0.52 (0.28-0.95) |  | 58/151974 | 0.66 (0.44-1.00) |  | 15/30626 | 0.57 (0.27-1.20) |  |  |
|  | Valine |  |  |  |  |  |  |  |  |  | 0.5923 |
|  | Low | 32/104369 | Reference | 0.3121 | 68/147385 | Reference | 0.0124 | 24/29384 | Reference | 0.0093 |  |
|  | Intermediate | 27/104752 | 0.63 (0.35-1.15) |  | 60/147773 | 0.82 (0.56-1.21) |  | 18/29406 | 0.64 (0.33-1.23) |  |  |
|  | High | 27/108452 | 0.74 (0.41-1.33) |  | 51/151919 | 0.57 (0.37-0.89) |  | 13/30556 | 0.37 (0.18-0.79) |  |  |
| Parkinson's Disease | Isoleucine |  |  |  |  |  |  |  |  |  | 0.6051 |
|  | Low | 45/104658 | Reference | 0.4943 | 69/147609 | Reference | 0.6017 | 13/29404 | Reference | 0.7676 |  |
|  | Intermediate | 50/104295 | 0.97 (0.63-1.50) |  | 86/147261 | 1.10 (0.78-1.56) |  | 21/29363 | 1.49 (0.72-3.07) |  |  |
|  | High | 63/108198 | 1.15 (0.75-1.75) |  | 100/151663 | 1.10 (0.78-1.57) |  | 21/30531 | 1.16 (0.54-2.50) |  |  |
|  | Leucine |  |  |  |  |  |  |  |  |  | 0.3027 |
|  | Low | 48/104155 | Reference | 0.9583 | 78/147211 | Reference | 0.737 | 8/29440 | Reference | 0.244 |  |
|  | Intermediate | 50/104664 | 0.90 (0.59-1.39) |  | 67/147643 | 0.69 (0.48-0.99) |  | 26/29270 | 3.38 (1.45-7.89) |  |  |
|  | High | 60/108332 | 1.00 (0.65-1.54) |  | 110/151680 | 1.01 (0.72-1.42) |  | 21/30588 | 2.02 (0.81-5.02) |  |  |
|  | Valine |  |  |  |  |  |  |  |  |  | 0.0717 |
|  | Low | 51/104206 | Reference | 0.4224 | 71/147284 | Reference | 0.9336 | 10/29409 | Reference | 0.6512 |  |
|  | Intermediate | 50/104632 | 0.75 (0.49-1.15) |  | 84/147605 | 0.93 (0.65-1.31) |  | 28/29353 | 2.50 (1.15-5.40) |  |  |
|  | High | 57/108314 | 0.83 (0.54-1.27) |  | 100/151645 | 1.01 (0.71-1.43) |  | 17/30536 | 1.36 (0.58-3.21) |  |  |

Model 3 was adjusted for age, sex, ethnicity, income, work, education, drink, smoke, BMI, SBP, hypertension, diabetes, stroke, HDL, LDL, TG, TC.

**Supplementary Table 8**. Association of branched-chain amino acids (BCAAs) with Dementia, Alzheimer’s disease and Parkinson’s disease after further Alzheimer's Disease adjusting for other covariates

| BCAA | No. Dementia cases/person-years | Dementia HR (95% CI) | | | No. Alzheimer’s Disease cases/  person-years | Alzheimer’s Disease HR (95% CI) | | | No. Parkinson’s  disease cases/  person-years | Parkinson’s Disease HR (95% CI) | | |
| --- | --- | --- | --- | --- | --- | --- | --- | --- | --- | --- | --- | --- |
|  |  | Model 1 | Model 2 | Model 3 |  | Model 1 | Model 2 | Model 3 |  | Model 1 | Model 2 | Model 3 |
| Isoleucine |  |  |  |  |  |  |  |  |  |  |  |  |
| Q1 | 442/283911 | Reference | Reference | Reference | 119/284683 | Reference | Reference | Reference | 134/284458 | Reference | Reference | Reference |
| Q2 | 433/283288 | 0.84 (0.74-0.97) | 0.84 (0.73-0.96) | 0.84 (0.74-0.96) | 121/284023 | 0.95 (0.73-1.23) | 0.95 (0.74-1.23) | 0.95 (0.74-1.24) | 155/283699 | 1.01 (0.8-1.28) | 0.99 (0.78-1.25) | 0.99 (0.78-1.25) |
| Q3 | 455/284417 | 0.82 (0.71-0.95) | 0.83 (0.72-0.95) | 0.83 (0.72-0.95) | 80/285290 | 0.62 (0.46-0.85) | 0.63 (0.47-0.84) | 0.63 (0.47-0.84) | 179/284826 | 1.13 (0.89-1.43) | 1.07 (0.85-1.36) | 1.07 (0.85-1.35) |
| P for trend |  | 0.0069 | 0.0071 | 0.0068 |  | 0.0029 | 0.0020 | 0.0021 |  | 0.3131 | 0.5220 | 0.5272 |
| Leucine |  |  |  |  |  |  |  |  |  |  |  |  |
| Q1 | 481/282880 | Reference | Reference | Reference | 134/283696 | Reference | Reference | Reference | 136/283432 | Reference | Reference | Reference |
| Q2 | 404/284033 | 0.73 (0.64-0.84) | 0.73 (0.64-0.84) | 0.73 (0.64-0.84) | 97/284787 | 0.69 (0.52-0.9) | 0.7 (0.53-0.91) | 0.7 (0.53-0.91) | 146/284530 | 0.93 (0.73-1.18) | 0.91 (0.71-1.15) | 0.91 (0.71-1.15) |
| Q3 | 445/284702 | 0.74 (0.64-0.85) | 0.75 (0.66-0.86) | 0.74 (0.65-0.85) | 89/285514 | 0.63 (0.47-0.85) | 0.64 (0.48-0.85) | 0.64 (0.48-0.85) | 186/285021 | 1.12 (0.88-1.42) | 1.07 (0.85-1.35) | 1.06 (0.84-1.35) |
| P for trend |  | 0.0001 | 0.0001 | 0.0001 |  | 0.0017 | 0.0018 | 0.0016 |  | 0.3132 | 0.4976 | 0.5174 |
| Valine |  |  |  |  |  |  |  |  |  |  |  |  |
| Q1 | 449/283144 | Reference | Reference | Reference | 126/283867 | Reference | Reference | Reference | 134/283614 | Reference | Reference | Reference |
| Q2 | 414/284075 | 0.77 (0.67-0.88) | 0.77 (0.67-0.88) | 0.77 (0.67-0.88) | 104/284818 | 0.74 (0.56-0.96) | 0.76 (0.58-0.98) | 0.75 (0.58-0.98) | 164/284478 | 1.05 (0.83-1.33) | 1.02 (0.81-1.29) | 1.02 (0.81-1.28) |
| Q3 | 467/284396 | 0.77 (0.66-0.89) | 0.78 (0.68-0.9) | 0.77 (0.67-0.89) | 90/285312 | 0.62 (0.46-0.84) | 0.63 (0.48-0.85) | 0.63 (0.47-0.84) | 170/284891 | 1.04 (0.81-1.34) | 0.97 (0.77-1.24) | 0.97 (0.76-1.23) |
| P for trend |  | 0.0004 | 0.0007 | 0.0004 |  | 0.0015 | 0.0017 | 0.0014 |  | 0.7609 | 0.8014 | 0.7815 |

Model 1 was adjusted for age, sex, ethnicity, income, work, education, drink, smoke, BMI, SBP, hypertension, diabetes;

Model 2 was adjusted for age, sex, ethnicity, income, work, education, drink, smoke, BMI, SBP, hypertension, diabetes, stroke;

Model 3 was adjusted for age, sex, ethnicity, income, work, education, drink, smoke, BMI, SBP, hypertension, diabetes, stroke, HDL, LDL, TG, TC.

P value for trend calculated treating the BCAAs (tertiles) as a continuous variable.

**Supplementary Table 9.** Association between BCAA and Dementia / Alzheimer's Disease / Parkinson's Disease in the subgroups stratified by age, bmi, drinking status, education, and smoking in model 3.

| Disease | BCAAs | Stratified by age | | | | P for Interaction |
| --- | --- | --- | --- | --- | --- | --- |
|  |  | ＜60 years | | ≥60 years | |  |
|  |  | HR (95% CI) | P for trend | HR (95% CI) | P for trend |  |
| Dementia | Isoleucine |  |  |  |  | 0.1394 |
|  | Low | Reference | 0.4396 | Reference | 0.1652 |  |
|  | Intermediate | 0.70(0.50-0.98) |  | 0.87 (0.71 - 1.06) |  |  |
|  | High | 0.86(0.63-1.18) |  | 0.86 (0.71 - 1.06) |  |  |
|  | Leucine |  |  |  |  | 0.2622 |
|  | Low | Reference | 0.0943 | Reference | 0.0027 |  |
|  | Intermediate | 0.60 (0.43 - 0.85) |  | 0.70 (0.57 - 0.85) |  |  |
|  | High | 0.74 (0.54 - 1.02) |  | 0.73 (0.6 - 0.89) |  |  |
|  | Valine |  |  |  |  | 0.4051 |
|  | Low | Reference | 0.1558 | Reference | 0.0056 |  |
|  | Intermediate | 0.80 (0.57 - 1.11) |  | 0.75 (0.62 - 0.92) |  |  |
|  | High | 0.78 (0.56 - 1.09) |  | 0.74 (0.61 - 0.91) |  |  |
| Alzheimer's Disease | Isoleucine |  |  |  |  | 0.6798 |
|  | Low | Reference | 0.0338 | Reference | 0.051 |  |
|  | Intermediate | 0.66 (0.33 - 1.34) |  | 0.80 (0.50 - 1.27) |  |  |
|  | High | 0.43 (0.19 - 0.95) |  | 0.61 (0.37 - 1.00) |  |  |
|  | Leucine |  |  |  |  | 0.2126 |
|  | Low | Reference | 0.0051 | Reference | 0.0102 |  |
|  | Intermediate | 0.33 (0.15 - 0.73) |  | 0.58 (0.36 - 0.93) |  |  |
|  | High | 0.35 (0.16 - 0.75) |  | 0.53 (0.32 - 0.87) |  |  |
|  | Valine |  |  |  |  | 0.1472 |
|  | Low | Reference | 0.0047 | Reference | 0.0004 |  |
|  | Intermediate | 0.42 (0.20 - 0.89) |  | 0.55 (0.35 - 0.88) |  |  |
|  | High | 0.33 (0.15 - 0.74) |  | 0.41 (0.24 - 0.68) |  |  |
| Parkinson's Disease | Isoleucine |  |  |  |  | 0.5022 |
|  | Low | Reference | 0.564 | Reference | 0.3674 |  |
|  | Intermediate | 0.85 (0.53 - 1.35) |  | 1.11 (0.81 - 1.51) |  |  |
|  | High | 0.87 (0.55 - 1.38) |  | 1.16 (0.85 - 1.57) |  |  |
|  | Leucine |  |  |  |  | 0.084 |
|  | Low | Reference | 0.1894 | Reference | 0.8309 |  |
|  | Intermediate | 0.72 (0.45 - 1.14) |  | 0.96 (0.71 - 1.31) |  |  |
|  | High | 0.72 (0.45 - 1.16) |  | 1.03 (0.76 - 1.40) |  |  |
|  | Valine |  |  |  |  | 0.1094 |
|  | Low | Reference | 0.0812 | Reference | 0.4043 |  |
|  | Intermediate | 0.90 (0.58 - 1.41) |  | 1.05 (0.78 - 1.41) |  |  |
|  | High | 0.65 (0.40 - 1.06) |  | 0.88 (0.65 - 1.21) |  |  |

Model 3 was adjusted for age, sex, ethnicity, income, work, education, drink, smoke, BMI, SBP, hypertension, diabetes, stroke, HDL, LDL, TG, TC.

**Supplementary Table 10.** Relationship between BCAA and Dementia / Alzheimer's Disease / Parkinson's Disease in the subgroups stratified by ApoE.

| Disease | BCAAs | Stratified by ApoE | | | | P for Interaction |
| --- | --- | --- | --- | --- | --- | --- |
|  |  | Non-APOE ε4 carriers | | APOE ε4 carriers | |  |
|  |  | HR (95% CI) | P for trend | HR (95% CI) | P for trend |  |
| Dementia | Isoleucine |  |  |  |  | 0.5364 |
|  | Low | Reference | 0.1652 | Reference | 0.0677 |  |
|  | Intermediate | 0.87 (0.71 - 1.06) |  | 0.91 (0.73 - 1.13) |  |  |
|  | High | 0.86 (0.71 - 1.06) |  | 0.81 (0.64 - 1.02) |  |  |
|  | Leucine |  |  |  |  | 0.5868 |
|  | Low | Reference | 0.0027 | Reference | 0.0249 |  |
|  | Intermediate | 0.70 (0.57 - 0.85) |  | 0.76 (0.61 - 0.95) |  |  |
|  | High | 0.73 (0.60 - 0.89) |  | 0.77 (0.61 - 0.97) |  |  |
|  | Valine |  |  |  |  | 0.8619 |
|  | Low | Reference | 0.0056 | Reference | 0.0505 |  |
|  | Intermediate | 0.75 (0.62 - 0.92) |  | 0.9 (0.72 - 1.12) |  |  |
|  | High | 0.74 (0.61 - 0.91) |  | 0.79 (0.62 - 1) |  |  |
| Alzheimer's Disease | Isoleucine |  |  |  |  | 0.9596 |
|  | Low | Reference | 0.051 | Reference | 0.0451 |  |
|  | Intermediate | 0.80 (0.50 - 1.27) |  | 1 (0.7 - 1.43) |  |  |
|  | High | 0.61 (0.37 - 1.00) |  | 0.65 (0.43 - 0.98) |  |  |
|  | Leucine |  |  |  |  | 0.9435 |
|  | Low | Reference | 0.0102 | Reference | 0.0854 |  |
|  | Intermediate | 0.58 (0.36 - 0.93) |  | 0.69 (0.47 - 1) |  |  |
|  | High | 0.53 (0.32 - 0.87) |  | 0.72 (0.48 - 1.06) |  |  |
|  | Valine |  |  |  |  | 0.3505 |
|  | Low | Reference | 0.0004 | Reference | 0.2098 |  |
|  | Intermediate | 0.55 (0.35 - 0.88) |  | 0.98 (0.68 - 1.42) |  |  |
|  | High | 0.41 (0.24 - 0.68) |  | 0.76 (0.51 - 1.15) |  |  |
| Parkinson's Disease | Isoleucine |  |  |  |  | 0.3992 |
|  | Low | Reference | 0.3674 | Reference | 0.6597 |  |
|  | Intermediate | 1.11 (0.81 - 1.51) |  | 0.87 (0.56 - 1.36) |  |  |
|  | High | 1.16 (0.85 - 1.57) |  | 0.9 (0.58 - 1.4) |  |  |
|  | Leucine |  |  |  |  | 0.4605 |
|  | Low | Reference | 0.8309 | Reference | 0.6448 |  |
|  | Intermediate | 0.96 (0.71 - 1.31) |  | 0.8 (0.5 - 1.27) |  |  |
|  | High | 1.03 (0.76 - 1.40) |  | 1.08 (0.69 - 1.68) |  |  |
|  | Valine |  |  |  |  | 0.5156 |
|  | Low | Reference | 0.4043 | Reference | 0.5862 |  |
|  | Intermediate | 1.05 (0.78 - 1.41) |  | 0.89 (0.56 - 1.4) |  |  |
|  | High | 0.88 (0.65 - 1.21) |  | 1.12 (0.71 - 1.75) |  |  |

Model 3 was adjusted for age, sex, ethnicity, income, work, education, drink, smoke, BMI, SBP, hypertension, diabetes, stroke, HDL, LDL, TG, TC.

**Supplementary Table 11.** Association between BCAA and Dementia, Alzheimer’s disease, Parkinson’s disease, excluding cases in the 2 years before follow-up.

| BCAA | No. Dementia cases/person-years | Dementia HR (95% CI) | | | No. Alzheimer’s Disease cases/person-years | Alzheimer’s Disease HR (95% CI) | | | No. Parkinson’s Disease cases/person-years | Parkinson’s Disease HR (95% CI) | | |
| --- | --- | --- | --- | --- | --- | --- | --- | --- | --- | --- | --- | --- |
|  |  | Model 1 | Model 2 | Model 3 |  | Model 1 | Model 2 | Model 3 |  | Model 1 | Model 2 | Model 3 |
| Isoleucine |  |  |  |  |  |  |  |  |  |  |  |  |
| Q1 | 430/280867 | Reference | Reference | Reference | 117/281636 | Reference | Reference | Reference | 131/281344 | Reference | Reference | Reference |
| Q2 | 420/280491 | 0.98 (0.86-1.12) | 0.87 (0.76-1) | 0.84 (0.73-0.96) | 116/281169 | 0.99 (0.77-1.29) | 0.93 (0.71-1.2) | 0.92 (0.71-1.2) | 152/280848 | 1.16 (0.92-1.47) | 0.97 (0.76-1.23) | 0.99 (0.78-1.25) |
| Q3 | 458/289898 | 1.03 (0.9-1.17) | 0.91 (0.8-1.04) | 0.83 (0.73-0.96) | 84/290853 | 0.69 (0.52-0.92) | 0.67 (0.5-0.89) | 0.66 (0.49-0.88) | 182/290278 | 1.35 (1.08-1.69) | 1.08 (0.86-1.36) | 1.1 (0.87-1.39) |
| P for trend |  | 0.6659 | 0.1965 | 0.0114 |  | 0.0141 | 0.0057 | 0.0053 |  | 0.0092 | 0.4553 | 0.4019 |
| Leucine |  |  |  |  |  |  |  |  |  |  |  |  |
| Q1 | 466/280006 | Reference | Reference | Reference | 129/280768 | Reference | Reference | Reference | 135/280480 | Reference | Reference | Reference |
| Q2 | 387/281029 | 0.83 (0.72-0.94) | 0.75 (0.65-0.85) | 0.72 (0.63-0.83) | 88/281712 | 0.67 (0.51-0.87) | 0.65 (0.49-0.85) | 0.66 (0.5-0.88) | 143/281438 | 1.05 (0.83-1.33) | 0.88 (0.69-1.11) | 0.9 (0.71-1.14) |
| Q3 | 455/290221 | 0.94 (0.82-1.07) | 0.83 (0.73-0.95) | 0.76 (0.67-0.88) | 98/291032 | 0.72 (0.55-0.93) | 0.71 (0.54-0.94) | 0.72 (0.55-0.95) | 187/290553 | 1.34 (1.07-1.67) | 1.04 (0.83-1.31) | 1.06 (0.84-1.34) |
| P for trend |  | 0.3222 | 0.0098 | 0.0002 |  | 0.0150 | 0.0165 | 0.0153 |  | 0.0088 | 0.6167 | 0.5193 |
| Valine |  |  |  |  |  |  |  |  |  |  |  |  |
| Q1 | 433/280268 | Reference | Reference | Reference | 122/280934 | Reference | Reference | Reference | 132/280673 | Reference | Reference | Reference |
| Q2 | 406/280981 | 0.93 (0.81-1.07) | 0.81 (0.7-0.92) | 0.78 (0.68-0.89) | 102/281682 | 0.83 (0.64-1.08) | 0.75 (0.57-0.98) | 0.76 (0.58-0.99) | 161/281327 | 1.22 (0.97-1.53) | 0.99 (0.79-1.25) | 1.02 (0.81-1.29) |
| Q3 | 469/290007 | 1.04 (0.91-1.19) | 0.88 (0.77-1) | 0.79 (0.68-0.9) | 91/290896 | 0.72 (0.55-0.94) | 0.65 (0.49-0.85) | 0.64 (0.48-0.86) | 172/290470 | 1.26 (1-1.58) | 0.97 (0.77-1.22) | 0.99 (0.78-1.25) |
| P for trend |  | 0.5281 | 0.0734 | 0.0010 |  | 0.0154 | 0.0020 | 0.0025 |  | 0.0515 | 0.7623 | 0.8992 |

Model 1 was adjusted for age, sex, ethnicity, income, work, education, drink, smoke, BMI, SBP, hypertension, diabetes;

Model 2 was adjusted for age, sex, ethnicity, income, work, education, drink, smoke, BMI, SBP, hypertension, diabetes, stroke;

Model 3 was adjusted for age, sex, ethnicity, income, work, education, drink, smoke, BMI, SBP, hypertension, diabetes, stroke, HDL, LDL, TG, TC.

P value for trend calculated treating the BCAAs (tertiles) as a continuous variable.
